# Purification of Crimean–Congo hemorrhagic fever virus nucleoprotein and its utility for serological diagnosis

**DOI:** 10.1038/s41598-021-81752-0

**Published:** 2021-01-27

**Authors:** Boniface Pongombo Lombe, Hiroko Miyamoto, Takeshi Saito, Reiko Yoshida, Rashid Manzoor, Masahiro Kajihara, Masayuki Shimojima, Shuetsu Fukushi, Shigeru Morikawa, Tomoki Yoshikawa, Takeshi Kurosu, Masayuki Saijo, Qing Tang, Justin Masumu, David Hawman, Heinz Feldmann, Ayato Takada

**Affiliations:** 1 Division of Global Epidemiology, Hokkaido University Research Center for Zoonosis Control, Sapporo, Japan; 2 Central Veterinary Laboratory of Kinshasa, Kinshasa, Democratic Republic of the Congo; 3 Faculty of Veterinary Medicine, National Pedagogic University, Kinshasa, Democratic Republic of the Congo; 4 Department of Virology 1, National Institute of Infectious Diseases, Tokyo, Japan; 5 National Institute for Viral Disease Control and Prevention, Institute of Infectious Diseases Control and Prevention, Chinese Center for Disease Control and Prevention, Beijing, China; 6 Institut National de Recherche Biomédicale, Kinshasa, Democratic Republic of the Congo; 7 Laboratory of Virology, Division of Intramural Research, National Institute of Allergy and Infectious Diseases, National Institutes of Health, Rocky Mountain Laboratories, Hamilton, USA; 8 Department of Disease Control, School of Veterinary Medicine, The University of Zambia, Lusaka, Zambia; 9 Global Institution for Collaborative Research and Education, Global Station for Zoonosis Control, Hokkaido University, Sapporo, Japan; 10 Present Address: Faculty of Veterinary Medicine, Okayama University of Science, Okayama, Japan

**Keywords:** Infectious-disease diagnostics, Virology

## Abstract

Crimean–Congo hemorrhagic fever virus (CCHFV) causes a zoonotic disease, Crimean–Congo hemorrhagic fever (CCHF) endemic in Africa, Asia, the Middle East, and Southeastern Europe. However, the prevalence of CCHF is not monitored in most of the endemic countries due to limited availability of diagnostic assays and biosafety regulations required for handling infectious CCHFV. In this study, we established a protocol to purify the recombinant CCHFV nucleoprotein (NP), which is antigenically highly conserved among multiple lineages/clades of CCHFVs and investigated its utility in an enzyme-linked immunosorbent assay (ELISA) to detect CCHFV-specific antibodies. The NP gene was cloned into the pCAGGS mammalian expression plasmid and human embryonic kidney 293 T cells were transfected with the plasmid. The expressed NP molecule was purified from the cell lysate using cesium-chloride gradient centrifugation. Purified NP was used as the antigen for the ELISA to detect anti-CCHFV IgG. Using the CCHFV NP-based ELISA, we efficiently detected CCHFV-specific IgG in anti-NP rabbit antiserum and CCHFV-infected monkey serum. When compared to the commercially available Blackbox CCHFV IgG ELISA kit, our assay showed equivalent performance in detecting CCHFV-specific IgG in human sera. These results demonstrate the usefulness of our CCHFV NP-based ELISA for seroepidemiological studies.

## Introduction

Crimean–Congo hemorrhagic fever virus (CCHFV) is an enveloped and segmented negative-sense RNA virus belonging to the genus *Orthonairovirus*, family *Nairoviridae*, order *Bunyavirales*. Its genome consists of L, M, and S segments encoding RNA-dependent RNA polymerase, glycoproteins Gn/Gc, and nucleoprotein (NP), respectively^1,2^. CCHFV causes a zoonotic arbovirosis of medical significance, Crimean–Congo hemorrhagic fever (CCHF), endemic in Africa, Asia, the Middle East, and Southeastern Europe^3,4^. CCHF is the most widely distributed tick-born zoonosis, as demonstrated by the detection of the virus and virus-specific antibodies in over 57 countries, expanding continually its geographic distribution linked to the habitat of its reservoir/primary vector-host, *Hyalomma* ticks. CCHFV also infects domestic animals, wildlife, and birds^5,6^. Although CCHFV infection of these animals and birds, despite high viremia, is clinically asymptomatic, they play a role as potential sources of human CCHF, which is linked to a public health warning^6–9^.

In most of the endemic countries, CCHFV infection is not regularly monitored due to the limited availability of diagnostic assays and it is often identified in the wake of nosocomial infections. CCHFV is one of the World Health Organization priority pathogens needing urgent research and development, with attention to diagnostic tools, to ensure preparedness for potential outbreaks^10^. However, most of the existing diagnostic tools such as in-house assays are not readily available, and commercialized assays are not cost-effective for serosurveillance studies^7,10^. In addition, CCHFV requires the highest level of biocontainment (Biosafety level 4), hampering its handling for experimental studies. Thus, there are only a few laboratories that can handle infectious CCHFV for virus isolation and production of its whole native viral antigens for serological diagnostic assays that are expected to be less affected by genomic diversity of CCHFVs than genetic detection^7,8,10,11^.

Previous studies have shown that some of the CCHFV structural proteins including NP are the predominant components that are antigenically conserved among CCHFV strains^12–14^ and induce a high immune response^10,15^. Recombinant CCHFV NPs expressed in insect, bacterial, plant, and mammalian cells have been used as antigens for serological assays^13,16–18^. However, the expression and purification processes often affect its conformation, structure, and antigenicity^14,19–21^. Herein, we report a simple procedure for expression and purification of the recombinant CCHFV NP in mammalian cells and its utility as an antigen for host species-independent serological assays for detection of CCHFV-specific IgG in serum/plasma samples.

## Methods

### Plasmids and expression of CCHFV NP

CCHFV (strain IbAr10200) was propagated in Vero E6 cells in the BSL4 Laboratory of the Rocky Mountain Laboratories (RML), NIAID, NIH. RNA extraction was performed according to standard operating protocols approved by the RML Institutional Biosafety Committee (IBC). Extracted RNA was used to synthesize cDNA using a SuperScript III reverse transcriptase kit (Invitrogen). The full-length NP gene was amplified using specific primers and cloned into a mammalian expression vector, pCAGGS/MCS. The NP sequence was confirmed by Sanger sequencing with a 3130xl/Genetic Analyzer. The Nairobi sheep disease virus (NSDV) NP gene (GenBank Accession number: NC_034386.1) was synthesized in pUCFa vector (Fasmac CO., LTD) and similarly cloned into the pCAGGS/MCS vector. Human embryonic kidney (HEK) 293 T cells seeded at 3–3.5 × 10^5^ cells/ml were grown on a 12-well plate in Dulbecco’s Modified Eagle Medium (DMEM) supplemented with 10% heat-inactivated fetal bovine serum (FBS), 100 U/ml penicillin, and 0.1 mg/ml streptomycin at 37 °C in a 5% CO_2_ incubator for 24 h. The cells were transfected with the plasmid (1 µg) using a TransIT-LT1 Transfection Reagent (Mirus Bio LLC) and then incubated at 37 °C for 48 h. The cells were washed 3 times with phosphate-buffered saline (PBS), treated with 250 µl of a lysis buffer (150 mM NaCl, 5 mM EDTA pH 8.0, 50 mM Tris–HCl pH 8.0, 1.0% NP-40, 0.5% sodium deoxycholate, and 0.1% sodium dodecyl sulfate (SDS) with cOmplete, Mini, EDTA-free protease inhibitor (Roche Diagnostics) and incubated for 5 min on a swing rotator. The cell lysate was centrifuged at 12,000 rpm for 10 min at 4 °C. The supernatant was collected and used for SDS–polyacrylamide gel electrophoresis (SDS-PAGE) and Western blotting.

### Serum samples

A rabbit antiserum to CCHFV NP was used as a positive control serum^13^. A rabbit antiserum to Ebola virus (EBOV) NP^22^ was also used as a negative control rabbit serum. Mouse antisera were obtained by immunizing animals intraperitoneally with purified CCHFV and NSDV NPs (40–50 μg) twice at 4-week intervals followed by serum collection 2 weeks after the second immunization. CCHFV (Hoti)-infected and EBOV (Kikwit)-infected monkey sera were treated with gamma-irradiation according to the RML IBC-approved SOP before use^23^. These sera were collected from nonhuman primates using animal study protocols approved by the RML Animal Use and Care Committee. Studies were performed in compliance with the Animal Welfare Act and other relevant statutes and regulations relating to animals and experiments involving animals and adhered to the principles stated in the Guide for the Care and Use of Laboratory Animals, National Research Council, 2011. A panel of serum samples previously collected from CCHF-suspected patients during an outbreak in the Xinjiang Uygur Autonomous Region of China was used for validation of assays^24^. The sera used in the present study were collected under informed consent. In the case of unconscious patients and children less than 20 years of age, informed consent was obtained from their family members and parents, respectively. The use of these human sera was approved by the medical research ethics committee of the National Institute of Infectious Diseases for the use of human subjects, Tokyo, Japan (No. 10). All methods were carried out in accordance with relevant guidelines and regulations.

### Immunofluorescent assay

HEK293T cells were cultured for 24 h in a chambered cell culture slide glass coverslip (Thermo Fisher Scientific) pre-coated with Cultrex Poly-L-Lysine (Bio-techne) and then transfected with the NP-expression plasmid using TransIT-LT1 transfection reagent (Mirus Bio LLC). At 24 h post-transfection, cells were washed with cold PBS and fixed with 4% paraformaldehyde for 20 min. After washing with PBS, the cells were incubated for 30 min with PBS supplemented with 1% bovine serum albumin. As primary and secondary antibodies, rabbit antiserum to CCHFV NP and Alexa Fluor 448-labeled donkey anti-rabbit IgG (H + L) (Aurion Immuno Gold Reagents & Accessories) were used, respectively. The cells were also stained with 4′,6-diamidino-2-phenylindole, dihydrochloride (DAPI). They were analyzed by confocal microscopy (Zeiss LSM 780) for image acquisition and processing using Zen software (Carl Zeiss Microscopy GmbH).

### SDS-PAGE and Western blotting

Samples (i.e., cell lysates, fractions of cesium chloride [CsCl] gradient centrifugation, and purified NP) were mixed with Laemmli sample buffer (Bio-Rad) with 5% β-mercaptoethanol, boiled for 5 min, and then loaded for 12% SDS-PAGE, followed by Coomassie blue staining with Quick-CBB Plus (Fujifilm Wako Chemical Corporation) and Western blotting. For Western blotting, separated proteins were transferred onto a polyvinylidene membrane (Immobilon-P, Merck), followed by blocking with 3% skimmed milk in PBS. The membrane was then washed with PBS containing 0.05% Tween-20 (PBST) 3 times and soaked for 1 h in 1% skimmed milk in PBST containing the anti-CCHFV NP rabbit serum. After washing with PBST 3 times, the membrane was incubated for 1 h with horseradish peroxidase (HRPO)-conjugated goat anti-rabbit IgG antibodies (KPL), followed by washing with PBST 3 times. Bound proteins were visualized with a 3,3′,5,5′-tetramethyl-benzidine (TMB) liquid substrate system for membrane (Sigma).

### Purification of recombinant CCHFV NP

HEK293T cells were seeded at 3–3.5 × 10^5^ cells/ml on 10 cm dishes. Twenty-four hours later, the cells were transfected with 10 µg of the plasmid encoding the CCHFV NP gene using TransIT-LT1. Transfected cells were harvested at 48–72 h post-transfection and washed 3 times with cold PBS by centrifugation 2 times at 4 °C. The cells were resuspended with PBS and transferred into a 1.5 ml tube and pelleted. Then PBS was removed, and the cells were stored at − 80 °C until use. Frozen cells were thawed on ice for 10 min and treated with 600 µl of lysis buffer (10 mM Tris–HCl, pH 7.8, 0.15 M NaCl, 1.0 mM EDTA, and 0.25% NP-40) in the presence of Halt Protease Inhibitor Single Use Cocktail (Thermo Scientific). Cells were mixed by pipetting and then incubated at 4 °C for 30 min with a SCINICS Revolution Mixing rotator (RVM-101). The lysate was centrifuged at 12,000 rpm at 4 °C for 10 min and the supernatant was collected. The supernatant was then loaded on the top of 20–50% (w/v) discontinuous CsCl gradients in Tris-buffered saline (TBS) layered from the top to bottom in 1 ml volumes of 20, 30, 40, and 50% CsCl in a 5 ml centrifuge tube (Beckman), followed by high-speed centrifugation using a SW55Ti rotor in a BECKMAN COULTER Optima L-100 XP ultracentrifuge at 280,000 × *g* at 4 °C for 4 h. Fractions (500 µl) were collected from the top to bottom and then analyzed by SDS-PAGE and Western blotting to check for the presence of CCHFV NP and its purity in collected fractions. CCHFV NP fractions were pooled and diluted with TBS and pelleted by centrifugation 280,000 × *g* for 30–60 min as previously described^25^. The supernatant was discarded and CCHFV NP was resuspended with 100 µl PBS with Halt Protease Inhibitor (Thermo Fisher Scientific). The concentration of purified NP was measured using a Nanodrop ND 1000 spectrophotometer (Thermo Fisher Scientific). Purified NP was re-analyzed by SDS-PAGE and Western blotting. The purified CCHFV NP was then stored at -80 °C until use. For large-scale CCHFV NP purification, HEK293T cells were grown in a 150 × 25 mm dish. For purification, CsCl layers (2.2 ml each of 20, 30, 40, and 50%) in Ultra-Clear tubes (Beckman) and an SW41 rotor was used (210,000 × *g* at 4 °C for 15 h). Fractions (1 ml) were collected and NP fractions were pooled and pelleted at 210,000 × g for 2 h and processed as described above. NSDV NP was also purified in the same methods described above.

### Electron microscopy (EM)

Freshly purified NP was dialyzed against TBS overnight using an EasySep dialysis membrane MD-014–50 (Tomy Seiko) according to the manufacturer’s instructions. The protein was then concentrated using Amicon Ultra-0.5 mL Centrifugal Filters, Ultracel-3 K (Merck Millipore). Concentrated CCHFV NP samples (5 µl) were applied on a collodion-coated copper grid (Nisshin EM) for 5 min then the excess sample was absorbed using filter paper. A 20 µl drop of 2% uranyl acetate (UA) solution was applied for 10 min. The grid was then treated with new drops of UA solution 2 times for each 1 min. EM images were observed using a Hitachi H7650 transmission electron microscopy (TEM) system (Hitachi High Technology Corporation). For immunogold staining, the rabbit antiserum to CCHFV NP and 5 nm gold-labeled goat anti-rabbit IgG (Biorbyt LLC) were used, followed by 2% UA staining.

### Enzyme-linked immunosorbent assay (ELISA)

ELISA was performed as previously described^22^. Briefly, 96-well ELISA plates (Nunc Maxisorp) were coated with the purified recombinant NP antigen, inactivated whole viral antigens (gamma-irradiated supernatant from CCHFV-infected SW-13 cells), or mock supernatant (supernatant from mock-infected Vero E6 cells) overnight at 4 °C, followed by blocking with 3% skimmed milk in PBS, and incubated with primary antibodies (i.e., rabbit, monkey, or human sera) in PBST containing 2% FBS for 1 h at room temperature. Bound antibodies were visualized with HRPO-conjugated goat anti-rabbit IgG (H + L) (Rockland), goat anti-monkey IgG(γ) (Rockland), and goat anti-mouse IgG (H + L) (Jackson ImmunoResearch) antibodies or Purified Recomb Protein A/G (Thermo Fisher Scientific)^26^, and the TMB liquid substrate system for ELISA (Sigma). The reaction was stopped by adding 1 N phosphoric acid, and the optical density was measured. A commercially available ELISA-based serum diagnosis kit, Blackbox CCHFV IgG ELISA Kit (Blackbox-ELISA)^16^ (Diagnostic Development Laboratory, Bernhard Nocht Institute for Tropical Medicine) was used according to the manufacturer’s protocol. ELISA index values were calculated based on the values of optical density (OD) at 450 and 620 nm for both the CCHFV NP-based ELISA and Blackbox-ELISA according to the formula provided by the manufacturer.

## Results

### Expression and purification of CCHFV NPs

**Figure 1:**
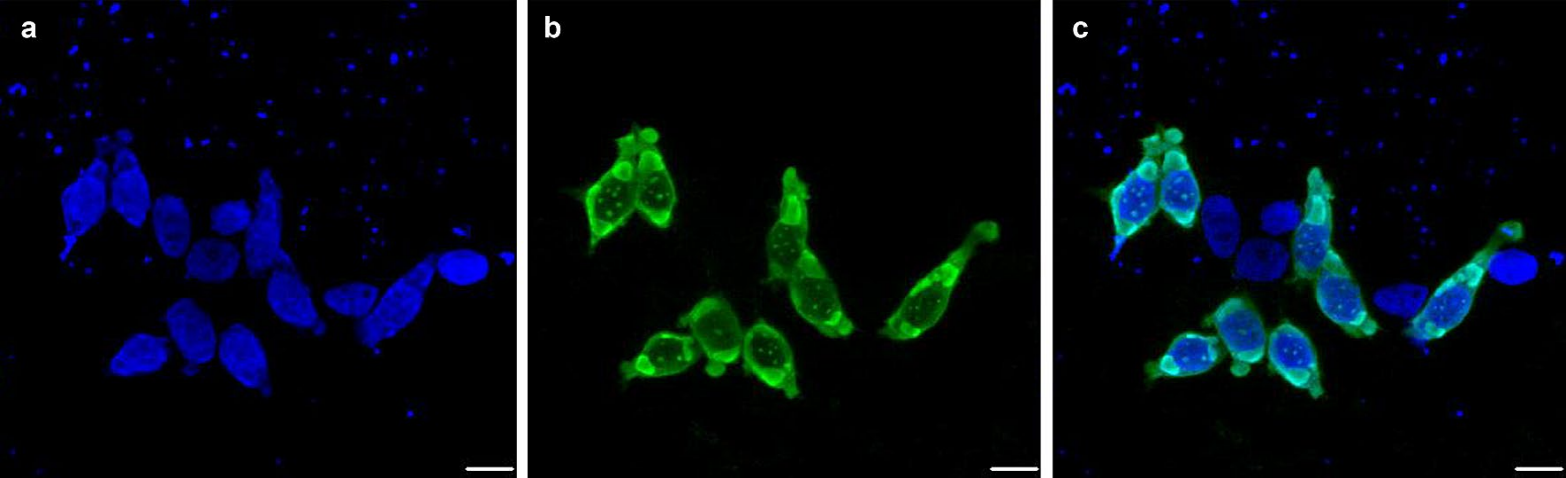
Cytoplasmic localization of expressed CCHFV NP. HEK293T cells transfected with the CCHFV NP-expressing plasmid were stained with DAPI (shown in blue) (**a**) and the rabbit antiserum to CCHFV NP followed by the donkey anti-rabbit IgG (H + L) antibody conjugated with Alexa Fluor 448 (shown in green) (**b**). Merged images of the stained cells are also shown (**c**). Scale bars represent 20 µm.

**Figure 2:**
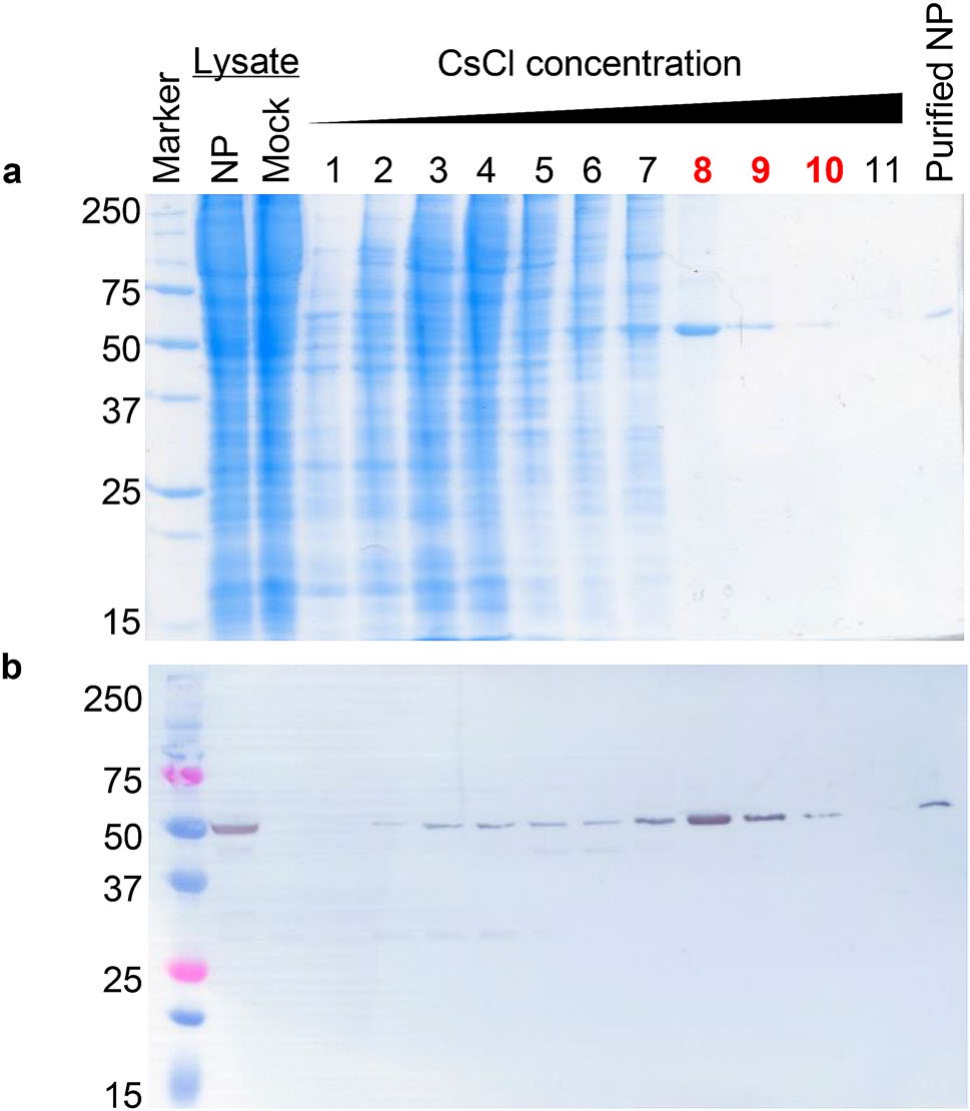
Purification of CCHFV NP by CsCl density gradient centrifugation. pCAGGS-CCHFV/NP-transfected HEK293T cells were lysed and fractionated through CsCl density gradient centrifugation as descibed in Methods. Each fraction was analyzed by SDS-PAGE (**a**) and Western blotting (**b**). Crude lysates of pCAGGS-CCHFV/NP- and mock-transfected HEK293T cells and purified NP obtained from pooled fractions 8–10 are also shown.

**Figure 3:**
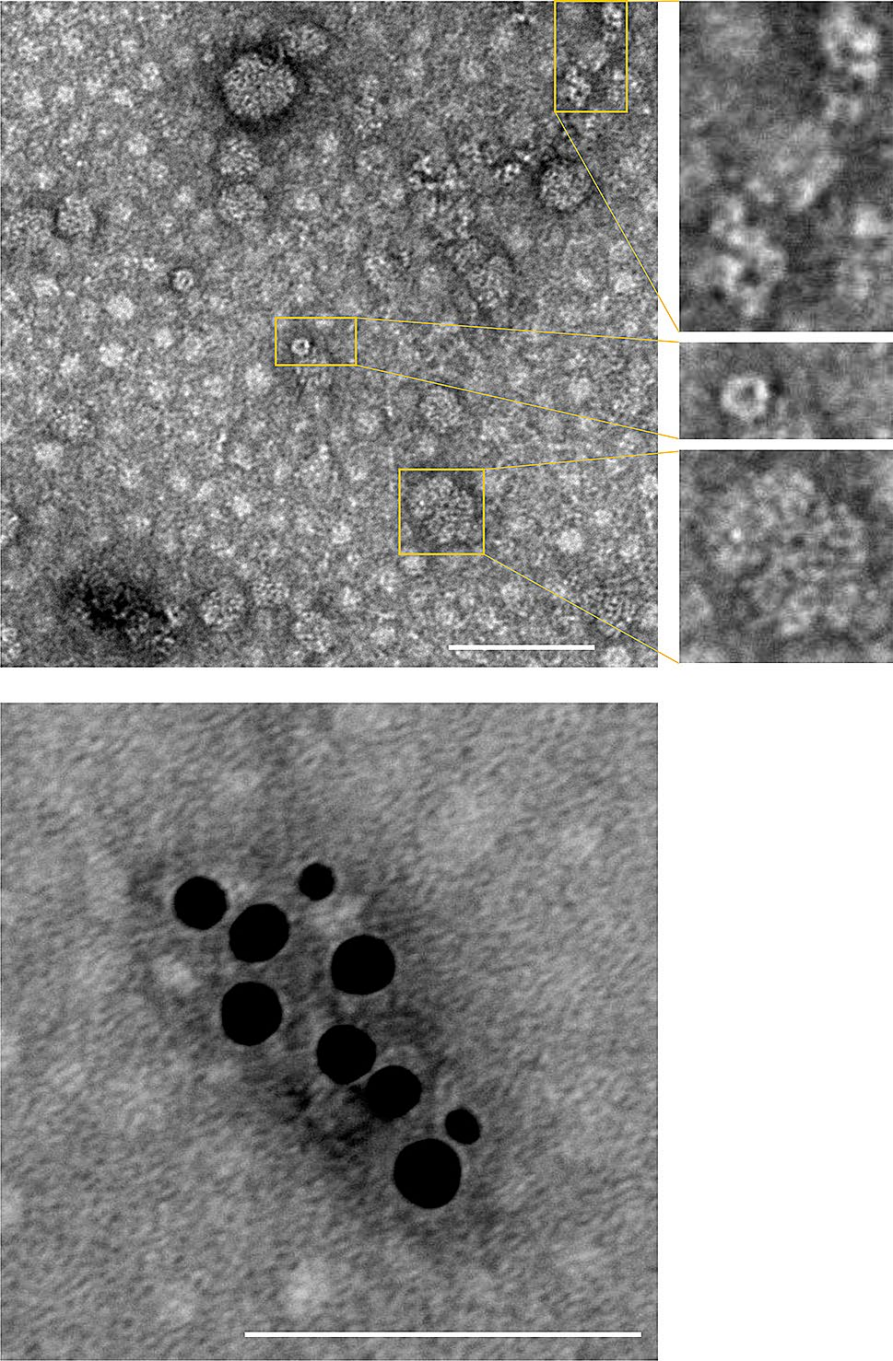
Electron micrographs of purified CCHFV NP. Purified CCHFV NP fractions were dialyzed overnight against TBS and then concentrated. Helical (upper right top panel), ring (upper right middle panel), and aggregated forms (upper right bottom panel) of the NP structures are shown. Polyclonal goat anti-rabbit IgG antibodies labeled with 5 nm gold particles were used for immunoelectron microscopy (bottom panel). TEM was operated at 80 kV, 1.0 µm, × 24.0 k magnification. Scale bars represent 100 nm.

HEK293T cells were transfected with the pCAGGS plasmid (pCAGGS-CCHFV/NP) for expression of the recombinant CCHFV NP and the cellular expression of the recombinant NP was observed using immunofluorescence assays (Fig. 1). We confirmed that CCHFV NP was successfully expressed by the transfection and formed densely stained inclusion body-like structures in the cytoplasm. We then purified the NP molecule from the transfected cells by equilibrium density gradient centrifugation in CsCl solution as described in “Methods”. The presence of the NP molecules in each fraction obtained from the CsCl density gradient centrifugation was analyzed with SDS-PAGE followed by Coomassie blue staining and Western blotting. According to the electrophoretic mobility consistent with the expected molecular mass of CCHFV NP (about 52 kDa), we detected the NP band in almost all the fractions, suggesting the presence of multiple forms of the NP oligomer (Fig. 2). To observe the morphology of NP molecules in the fractions, we performed TEM using the NP-rich fractions (e.g., fraction #8 in Fig. 2). TEM micrographs of the protein revealed that these fractions contained NPs in its oligomeric form showing ring- and helical-shaped architectures and their aggregates^19^, suggesting NP-NP intermolecular interaction forming complex structures in the fractions (Fig. 3). We then pooled the NP-rich fractions (fractions #8, #9, and #10 in Fig. 2) and collected them as purified CCHFV NP as described in “Methods”. The average OD_260_/OD_280_ ratio of purified NP fractions from multiple rounds of purification was 2.2, suggesting the presence of nucleic acids bound to the NP molecules.

### Establishment of the purified recombinant CCHFV NP-based ELISA

**Figure 4:**
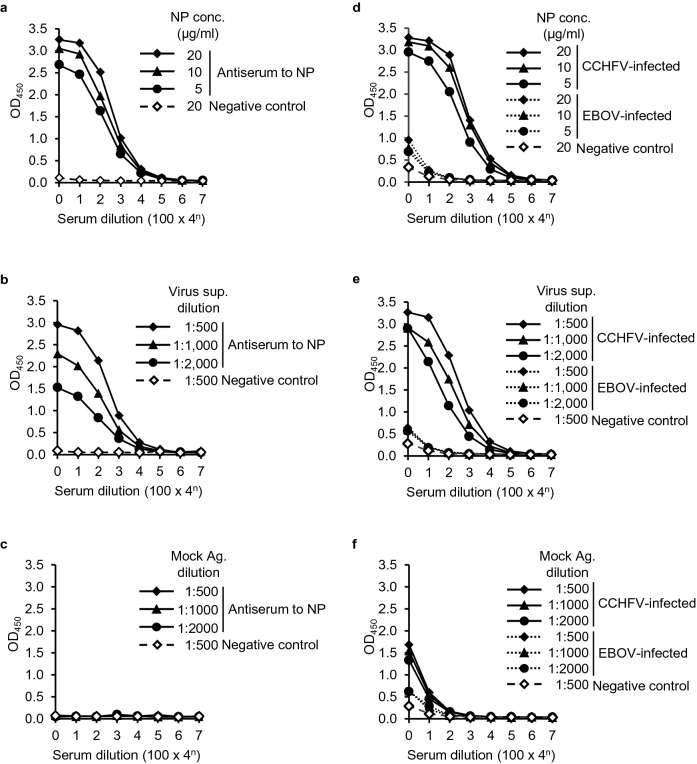
Detection of CCHFV NP-specific IgG in ELISA with purified CCHFV NP and whole CCHFV viral antigens. ELISA plates were coated with purified NP (5, 10, and 20 µg/ml) (**a**, **d**), serially diluted (1:500, 1:1,000, and 1:2,000) CCHFV-infected cell culture supernatant (Virus sup.) (**b**, **e**), or mock-infected supernatant (Mock Ag.) (**c**, **f**). Serial dilutions of the rabbit antiserum (**a**, **b**, **c**) and CCHFV-infected monkey serum (**d**, **e**, **f**) were used as primary antibodies, followed by detection with HRPO-conjugated anti-rabbit IgG and anti-monkey IgG antibodies, respectively. Negative control rabbit and monkey sera and EBOV-infected monkey serum were also tested.

We then used the purified CCHFV NP as an antigen for ELISA (5, 10, and 20 µg/ml) and investigated its utility to detect NP-specific IgG antibodies using the rabbit antiserum to CCHFV NP and CCHFV-infected monkey serum (Fig. 4a,d). The supernatants from CCHFV-infected Vero E6 cells and mock-infected supernatant were also used as positive and negative control antigens, respectively (Fig. 4b,c,e,f). In this experiment, host animal species-specific secondary antibodies (i.e., HRPO-conjugated anti-rabbit IgG and anti-monkey IgG antibodies) were used. We found that IgG antibodies to the CCHFV NP antigen, as well as to whole CCHFV antigen in the infected cell culture supernatant, were clearly detected in the rabbit antiserum and CCHFV-infected monkey serum but not in the EBOV-infected monkey serum and negative control sera. The endpoint antibody titers of the rabbit antiserum and the CCHFV-infected monkey serum to the purified NP antigen were 25,600–102,400. There was no remarkable difference in the obtained OD values between 10 and 20 μg/ml concentrations of the NP antigen, although the 5 μg/ml concentration of the antigen gave slightly lower OD values.

### Modification of the CCHFV NP-based ELISA using HRPO-conjugated protein A/G

**Figure. 5:**
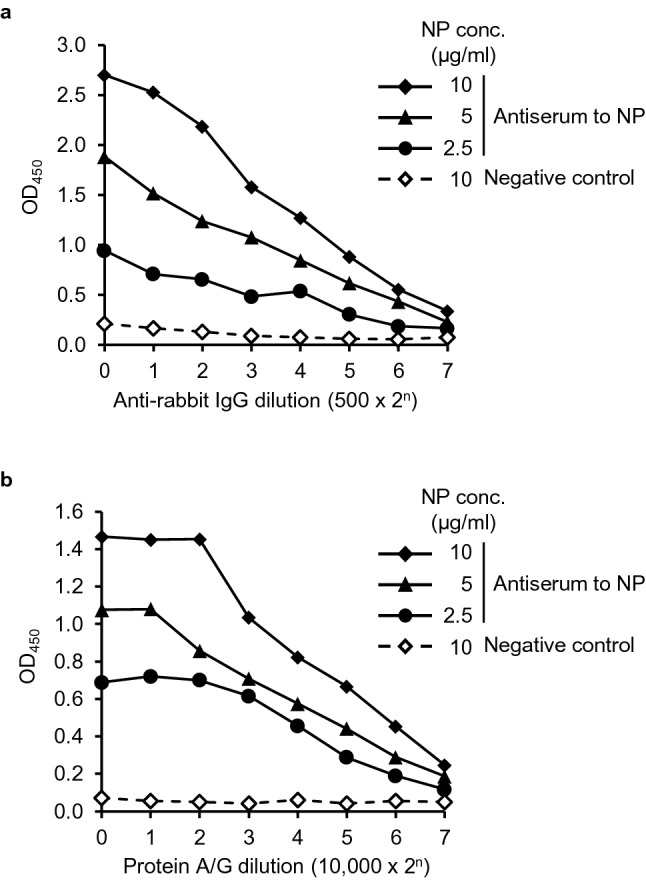
Detection of CCHFV NP-specific IgG using rabbit antiserum. ELISA plates were coated with the indicated concentrations (conc.) of purified NP and 1:1000 dilutions of the rabbit antiserum and negative control serum were used as primary antibodies. Serial dilutions of the HRPO-conjugated anti-rabbit IgG antibody (**a**) or the protein A/G reagent (**b**) were used for the detection of bound NP-specific antibodies.

To modify the CCHFV NP-based ELISA to detect NP-specific antibodies from a wide variety of animals, we examined the utility of HRPO-conjugated protein A/G in different conditions of ELISA using the rabbit antiserum (Fig. 5). We tested 3 different concentrations of the purified NP antigen (2.5, 5, and 10 μg/ml) with serial dilutions of HRPO-conjugated anti-rabbit IgG antibody (host-specific) or HRPO-conjugated protein A/G (species-independent) reagent to detect bound IgG antibodies in the rabbit antiserum. We found that 10 μg/ml gave the highest OD values for both HRPO-conjugated reagents and confirmed the dilution-dependent curves of the OD values, indicating that the HRPO-conjugated protein A/G reagent worked properly and could also be employed for the CCHFV NP-based ELISA. According to the curve trend, we used the antigen concentration of 10 μg/ml and 1:10,000 dilutions of HRPO-conjugated protein A/G, for the following experiment.

### The performance of the CCHFV NP-based ELISA compared to Blackbox-ELISA

**Figure 6:**
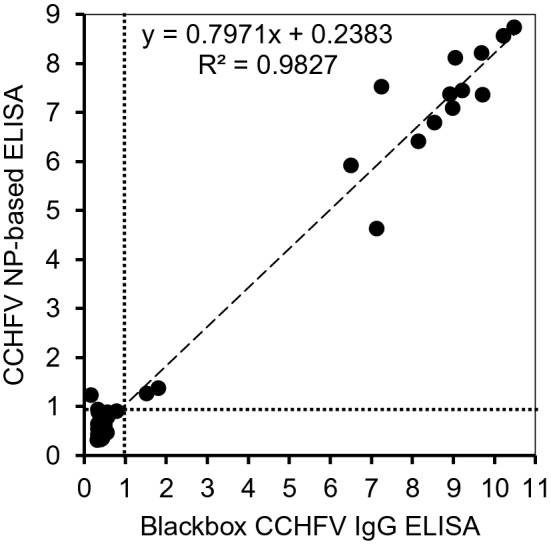
Correlation of IgG reactivity in human sera between the CCHFV NP-based ELISA and Blackbox-ELISA. ELISA plates were coated with purified NP (10 µg/ml). Serum samples were used at 1:100 dilution. The HRPO-conjugated protein A/G reagent (1:10,000 dilution) was used to detect bound antibodies. Scatter plots of the index values obtained by the CCHFV NP-based ELISA and Blackbox-ELISA are shown with the linear regression line (dashes). The coefficient of determination of R^2^ and the cutoff lines (dotted lines) of both the CCHFV NP-based ELISA and Blackbox-ELISA are also shown.

To further confirm the utility of our CCHFV NP-based IgG ELISA with the HRPO-conjugated protein A/G reagent, its performance was compared to a commercially available kit, Blackbox-ELISA, which can be used for human serum samples. Forty-five human serum samples collected during an outbreak in a known CCHFV endemic area were simultaneously tested using our CCHFV NP-based ELISA and Blackbox-ELISA. Among the 45 patient serum samples, 16 sera were previously defined as CCHFV IgG positives^24^. The newly established CCHFV NP-based ELISA detected all 16 of those positive samples, whereas Blackbox-ELISA detected only 15 of the positive samples (Table 1). We found that the OD index values obtained for the CCHFV NP-based ELISA and Blackbox ELISA showed a high positive correlation (correlation coefficient R^2^ = 0.98), indicating that these ELISA procedures had similar capacities to detect CCHFV NP-specific antibodies (Fig. 6). The sensitivity and specificity of our CCHFV NP-based ELISA were estimated by comparing the results to the Blackbox-ELISA and confidence intervals (CI) based on the Poisson-distribution approximation were calculated (Table 1). All 15 Blackbox-ELISA-positive samples were positive with the CCHFV NP-based IgG ELISA and most of the Blackbox-ELISA-negative samples (29/30) were also negative with CCHFV NP-based ELISA, which represented a sensitivity of 100% (15/15) (95% CI 78.20–100) and a specificity of 96.6% (29/30) (95% CI 82.78–99.92). Negative and positive predictive values were 100% (29/29) (95% CI 88.06–100) and 93.8% (15/16) (95% CI 69.76–99.84), respectively. The agreement rate with the Blackbox-ELISA was 97.8% (95% CI: 88.23–99.94).

**Table 1:** Performance of the CCHFV NP-based ELISA compared to Blackbox-ELISA.

		Blackbox-ELISA		
		+	−	Total
CCHFV NP-based ELISA	+	15	1	16
	−	0	29	29
	Total	15	30	45

Sensitivity: 100% (15/15) (95% CI 78.20–100).

Specificity: 96.6% (29/30) (95% CI 82.78–99.92).

Negative predictive value: 100% (29/29) (95% CI 88.06–100).

Positive predictive value: 93.8% (15/16) (95% CI 69.76–99.84).

Agreement rate: 97.8% (44/45) (95% CI 88.23–99.94).

### Limited cross-reactivity among antisera to CCHFV and NSDV NPs

**Figure 7:**
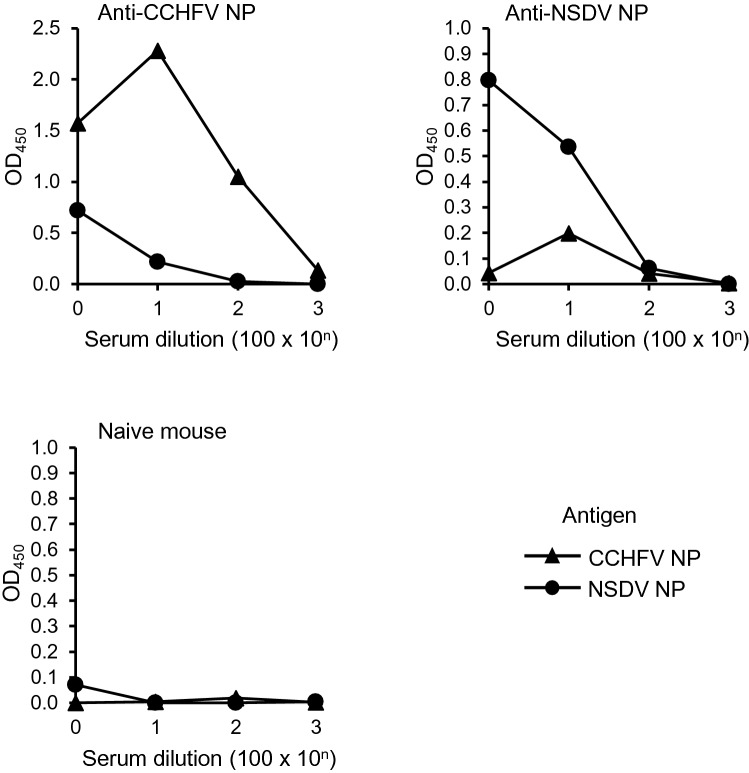
Cross-reactivity of antisera to CCHFV and NSDV NPs. ELISA plates were coated with 10 µg/ml of purified CCHFV NP, NSDV NP, or the mock antigen. Serial dilutions (1:100, 1:1,000, 1:10,000, and 1:100,000) of mouse antiserum to CCHFV, to NSDV NP, and naïve mouse serum were used as primary antibodies and incubated for 2 h at 4 °C. Peroxidase conjugate goat anti-mouse IgG (H + L) was used for detection of bound antibodies. To offset the nonspecific antibody reaction, the OD value of the mock antigen was subtracted for each dilution.

CCHFV patients and/or CCHFV-infected animals (e.g., sheep and goats) may have antibodies cross-reactive to NSDV, which is phylogenetically related to the CCHFV serogroup. Conversely, NSDV-infected animals may have cross-reactive antibodies to CCHFV. Thus, we produced mouse antisera to CCHFV and NSDV NPs and compared their reactivity to both antigens (Fig. 7). We found only a limited cross-reactivity among the antisera, suggesting that our CCHFV NP-based ELISA principally detected CCHFV-specific IgG antibodies.

## Discussion

Although CCHFV remains one of the priority pathogens needing urgent research and development of diagnostics, experimental studies involving live infectious CCHFV are restricted to the highest level biosafety containment laboratories around the world^10,11,16^. Alternatively, recombinant CCHFV proteins have been used to study their functions or to develop serological diagnostic tools. For example, CCHFV NP has been expressed in insect, bacterial, mammalian, and plant cells and used as a protein antigen to detect virus-specific antibodies^8,13,16,17,27,28^. However, accessibility to these serological diagnostic tools remains limited, probably because of the unavailability of the antigens or specific monoclonal antibodies for constant production and provision.

In this study, using the HEK293T cell line and pCAGGS plasmid, both of which are widely used for exogeneous protein expression in a large number of laboratories, we successfully expressed and purified full-length CCHFV NP. The cytoplasmic and granular (i.e., inclusion body-like) localization of CCHFV NP expressed in the cells was similar to that expressed in HeLa cells or CCHFV-infected Vero E6 cells^17,29^. Importantly, we used the untagged NP construct and purified it without affinity chromatography procedures, which generally require multiple steps that should be optimized depending on each laboratory condition to obtain a pure protein^20^. Thus far, NP constructs with a histidine-tag at the N- and/or C-terminal have been shown to be purified through affinity tag-based chromatography and used for diagnostic purposes^16,24,27,28^ or biological studies^30^. However, these methods are often unavailable for some laboratories due to cost and/or laboratory equipment issues. In addition, it is conceivable that untagged NP is antigenically more native than tagged forms and some conditions during the affinity purification process (e.g., low pH) might negatively affect the structure and function of NP^20^. Indeed, a CCHFV NP fusion protein containing a 6 × histidine-tag purified under denaturing conditions has been shown to be relatively unstable, although it can detect CCHFV IgG^21^. Thus, we believe that, compared to affinity tag-based purification, our method is a simpler and more effective purification procedure that enables us to obtain recombinant CCHFV NP that is conformationally close to authentic NP produced in CCHFV-infected cells.

The essential nature of NP is to bind nucleic acids and to form a ribonucleoprotein (RNP) complex. Both termini of the CCHFV S segment are involved conjointly in this essential function, RNA binding^30^. Like other viruses in the genus *Orthonairovirus*, CCHFV NP binds to nucleic acids and then undergoes a conformational change^19,31^. It was corroborated that his-tagged NPs purified by affinity chromatography with the OD_260_/OD_280_ ratios 1.3 and 1.49 had high-ordered oligomeric RNP structures that exhibited head and stalk domains^19,32^. When the RNP complex is formed, the stalk domain is thought to display a highly conserved epitope region that was suggested to be used for a universal CCHF diagnostic approach^13,14^. In the present study, the average ratio of OD_260_/OD_280_ (2.2) of the purified CCHFV NP was greater than those described previously, suggesting that it contained more RNA associated with NP molecules. This difference may be explained by the functionality of both termini (i.e., with or without tag sequences) to form the RNP complex. These observations suggest that untagged CCHFV NP purified through CsCl density gradient centrifugation is an NP-RNA complex that may expose the stalk domain more efficiently than tagged NP, which might result in increased ELISA cross-reactivity through the universal CCHFV NP stalk domain’s epitopes.

Commercially available CCHFV serological tools are specifically intended for the detection of human IgM or IgG (i.e., Blackbox CCHFV, VectoCrimean-CHF)^16,33^. On the other hand, serological surveillance of CCHFV infection in animals is also important and serves as an indicator of CCHF risk to humans^34,35^. Protein A/G is known to bind to multiple IgG classes of most mammalian species, including humans, domestic animals (cattle, goats, sheep, horses, rabbits, pigs, dogs, cats, alpacas, etc.), and wildlife. It has been widely used for purification of antibodies with affinity chromatography and, when conjugated with enzymes (e.g., HRPO), for routine immunoassays as an antibody detection tool^26,36–42^. In this study, we demonstrated the utility of the purified CCHFV NP antigen together with the HRPO-conjugated protein A/G reagent. This procedure has the advantages of simplicity and versatility. For species whose IgG affinity to protein A/G is not proven, host-specific secondary antibodies may remain useful.

It has been suggested that CCHFV NP is the most conserved viral protein among members of the *Nairoviridae* family^12–14^. It is noteworthy that NP of the IbAr10200 strain was recognized by antibodies in a monkey infected with another CCHFV strain, Hoti, belonging to a different clade from IbAr10200^43^. The detection of CCHFV NP-specific IgG in Chinese patients’ sera using one of the African strains, IbAr10200, also demonstrated serological cross-reactivity among genetically distinct strains/lineages, suggesting potential worldwide application of our CCHFV NP-based ELISA. On the other hand, it is important to pay attention to possible cross-reactivity of CCHFV NP antibodies to NSDV, which is a nairovirus in another serogroup, since some CCHFV-susceptible animals (e.g., sheep and goats) may have antibodies to this virus. However, we assume that there might be limited cross-reactivity since CCHFV NP was not recognized by antibodies directed against Dugbe virus belonging to the Nairobi sheep disease virus serogroup^44,45^. Indeed, our data also suggest that specificity of serum antibodies to CCHFV and NSDV could be distinguishable in NP-based ELISA. In future studies, the purified CCHFV NP antigen still has to be evaluated for IgM detection, which is important for clinical use during acute CCHFV infection.
